# Efficacy of priming and commitment posters on urgent care patients’ antibiotic expectations and knowledge: a cluster randomized trial

**DOI:** 10.1017/ash.2024.475

**Published:** 2025-01-06

**Authors:** Michael J. Cziner, Daniel E. Park, Rana F. Hamdy, Laura Rogers, Monique M. Turner, Cindy M. Liu

**Affiliations:** 1 Antibiotic Resistance Action Center, Department of Environmental and Occupational Health, Milken Institute School of Public Health, George Washington University, Washington, DC, USA; 2 Department of Epidemiology, New York University School of Global Public Health, New York, NY, USA; 3 Division of Infectious Diseases, Children’s National Hospital, Washington, DC, USA; 4 Department of Pediatrics, George Washington University School of Medicine and Health Sciences, Washington, DC, USA; 5 Department of Communication, Michigan State University, East Lansing, MI, USA

## Abstract

**Objective:**

Successfully educating urgent care patients on appropriate use and risks of antibiotics can be challenging. We assessed the conscious and subconscious impact various educational materials (informational handout, priming poster, and commitment poster) had on urgent care patients’ knowledge and expectations regarding antibiotics.

**Design:**

Stratified Block Randomized Control Trial.

**Setting:**

Urgent care centers (UCCs) in Colorado, Florida, Georgia, and New Jersey.

**Participants:**

Urgent care patients.

**Methods:**

We randomized 29 UCCs across six study arms to display specific educational materials (informational handout, priming poster, and commitment poster). The primary intention-to-treat (ITT) analysis evaluated whether the materials impacted patient knowledge or expectations of antibiotic prescribing by assigned study arm. The secondary as-treated analysis evaluated the same outcome comparing patients who recalled seeing the assigned educational material and patients who either did not recall seeing an assigned material or were in the control arm.

**Results:**

Twenty-seven centers returned 2,919 questionnaires across six study arms. Only 27.2% of participants in the intervention arms recalled seeing any educational materials. In our primary ITT analysis, no difference in knowledge or expectations of antibiotic prescribing was noted between groups. However, in the as-treated analysis, the handout and commitment poster were associated with higher antibiotic knowledge scores.

**Conclusions:**

Educational materials in UCCs are associated with increased antibiotic-related knowledge among patients when they are seen and recalled; however, most patients do not recall passively displayed materials. More emphasis should be placed on creating and drawing attention to memorable patient educational materials.

## Introduction

Antimicrobial resistance is among the greatest public health threats globally and nationally within the United States, posing significant threats to human, animal, and environmental health.^1,2^ Specifically, the Center for Disease Control and Prevention estimated that there were more than three million drug-resistant infections in the United States in 2019.^1^ Antimicrobial-resistant bacterial infections were associated with an estimated 4.95 million deaths worldwide in 2019.^3^

Antimicrobials, particularly antibiotics, are commonly prescribed in outpatient healthcare settings in the United States. There were 250 million oral antibiotic prescriptions in outpatient settings in the United States in 2018, equivalent to 763 prescriptions per 1000 US persons.^4^ Estimates suggest that 30%–56% of outpatient oral antibiotic prescriptions may be inappropriate.^5–8^ Antimicrobial stewardship programs are important and necessary in implementing interventions that target the prescribers. Providing education about appropriate use of antibiotics to patients themselves complements good antibiotic stewardship practice. Although studies suggest there is a discrepancy between provider perceptions and patient expectations of antibiotic prescriptions, medical providers continue to report that they perceive pressure to prescribe antibiotics from patients or parents of patients.^9–12^ Providers may fear that not prescribing antibiotics could drive patients away or garner negative reviews of their practice.^11^ This is especially worrisome for providers in urgent care settings, where patients may not have the same level of loyalty and trust in their urgent care provider compared to a longstanding provider with whom a patient has an established relationship.^11^ In this context, educating patients on the risks and dangers of taking unnecessary antibiotics may be particularly important. In addition to direct impacts on improving patient knowledge around antibiotics, providers may also be more likely to adhere to guidelines if they perceive that patient education materials are being effectively implemented in their clinical setting, thereby reducing unnecessary antibiotic prescribing.

Educating urgent care patients on appropriate use and risks of antibiotics remains challenging. Displaying educational materials, such as posters or handouts, is one technique that has been used to educate patients on medical issues. However, even within the larger scope of antibiotic-related patient education, this technique has varied in its effectiveness, with more memorable formats (eg GIFs, memes) being linked with increased effectiveness.^13–17^ Further, there have been a limited number of studies on patient education stewardship interventions specific to urgent care settings, with most being restricted to single urgent care networks.^18–20^

This study examined the effect of multiple educational materials on urgent care patients’ knowledge of antibiotic resistance and appropriate antibiotic use, as well as their expectation level for when an antibiotic prescription should be given. This study also compared outcomes of participants who recalled seeing any educational materials to those who did not recall seeing any educational material. Our study aims to elucidate the impact of educational materials on knowledge about appropriate use of antibiotics and antibiotic resistance, and on patients’ expectations for antibiotic prescriptions in a geographically diverse set of urgent care centers.

## Methods

### Setting and participants

Urgent care centers (UCCs) (n = 29) from four UCC networks located in Colorado, Florida, Georgia, and New Jersey were recruited to participate in a cluster randomized trial. All of the UCCs served both adult and pediatric patients. UCCs were recruited through emails via an Urgent Care Association listserv from July through October 2018, and study questionnaires were administered from October 2018 through February 2019. Eligible participants were either adult patients or guardians of pediatric patients (ie, any adult that may have accompanied a child to a UCC visit). The study was approved by the George Washington University (GWU) Institutional Review Board (#051866).

### Intervention

The educational materials used in this study were developed by a professional marketing and public relations firm (CommunicateHealth, Inc, Rockville, MD) based on quantitative and qualitative research conducted by the research firm Strategies 360, Inc (Seattle, WA) and refined in focus groups of urgent care patients. Specifically, Strategies 360 conducted a four-day online bulletin board focus group (QualBoard) among 36 recent U.S. UCC patients in March 2017, including parents of children (n = 15) and individuals with low health literacy (n = 13). Further quantitative evaluations of educational materials were performed by Strategies 360 and GfK (Nuremberg, Germany) among 610 recent U.S. UCC patients using GfK’s KnowledgePanelTM in April 2017.

The three educational materials included in this study included a priming poster, an informational handout, and a commitment poster (Supplemental Figure S1). The participating clinic was instructed to display the priming poster and the handout in a prominent location on the waiting room and place this poster in a prominent location in the exam room. The effect of the priming poster is based on automatic attitude activation.^21^ The educational handout was designed to educate patients that antibiotics are not effective for viral infections and to preemptively answer patients’ questions. The commitment poster,^22^ signed by the practice’s providers, leverages behavioral psychology to enhance accountability for appropriate antibiotic prescribing by displaying a pledge from the providers to responsibly prescribe antibiotics.

### Randomization

The UCCs were randomly assigned to study arms using stratified block randomization. Clinics were grouped by UCC network during the block assignments to ensure different arms were represented in each network. There was one control arm (ie, no materials displayed) and five intervention arms assigned using a 3 (commitment poster, priming poster, no poster) by 2 (handout and no handout) study design, resulting in three intervention arms with one material and two arms with two materials. This design was intended to assess the additive or multiplicative effect of two interventions compared with one intervention.

### Data collection

Patients completed a voluntary and anonymous study questionnaire at the end of their UCC visit. Due to resource limitations, the number of eligible patients who declined participation in the study was not tracked. The questionnaire included cued recall questions with three false images and three educational images from the study materials, participant knowledge regarding antibiotics, and their expectations of receiving antibiotics. Participant antibiotic knowledge included four questions that assessed information from the study handout (Supplemental Materials).

Patient antibiotic expectations were assessed on the question “What do you expect an antibiotic for?” regarding nine conditions: assessed cough, cold, virus, strep throat, flu, urinary tract infections, bronchitis, skin infection, and sinus infection. The expectation score represented a behavioral indicator and was not intended as a measure of knowledge. Expectation for any condition – both appropriate and inappropriate – received one point, resulting in a score ranging from 0 to 9, with 0 representing least likely to expect antibiotics and 9 being most likely to expect antibiotics. In supplemental analyses, we assessed patient antibiotic expectations for antibiotic-inappropriate conditions (ie, cough, cold, virus, flu, and bronchitis), with a score from 0 to 5.

### Data analysis

Responses from participant surveys were manually entered by the GWU team. Both intention-to-treat (ITT) and as-treated analyses were performed. The ITT analysis evaluated whether educational material assignment at UCCs impacted participants’ knowledge or expectation scores. The as-treated analysis focused on whether correctly recalling the material significantly improved participants’ antibiotic expectation or knowledge scores compared to participants who either (i) did not correctly recall seeing the material assigned to their arm or (ii) were from a center assigned to the control arm. For the as-treated analysis, patients assigned to an arm with two assigned materials that recalled seeing both materials were excluded due to sample size limitations; however, if they only saw one material, they were reassigned into the relevant arm for that material.

Descriptive statistics for categorical variables were analyzed using χ^2^ tests, Wilcoxon Rank Sum tests, and Kruskal–Wallis tests as appropriate. Continuous dependent variables (scores) were analyzed using analysis of variance (ANOVA) tests. Linear mixed effect models, clustered by individual clinics, were employed and controlled for the participant’s gender, age group, ethnicity, race, and education level. Spearman correlations were used to quantify associations between knowledge and expectation scores. To detect a 7.5% difference on the knowledge and expectation scores, we estimated needing a sample size of 155 participants per arm, with five clusters per intervention, an intraclass correlation coefficient of 0.2, a two-sided 5% significance level, and 80% power. Statistical analysis was conducted using SAS 9.4 (Cary, NC) and R (R version 4.3.2). The CONSORT checklist was utilized when writing this report.^23^

## Results

### Study population

**Figure 1.**
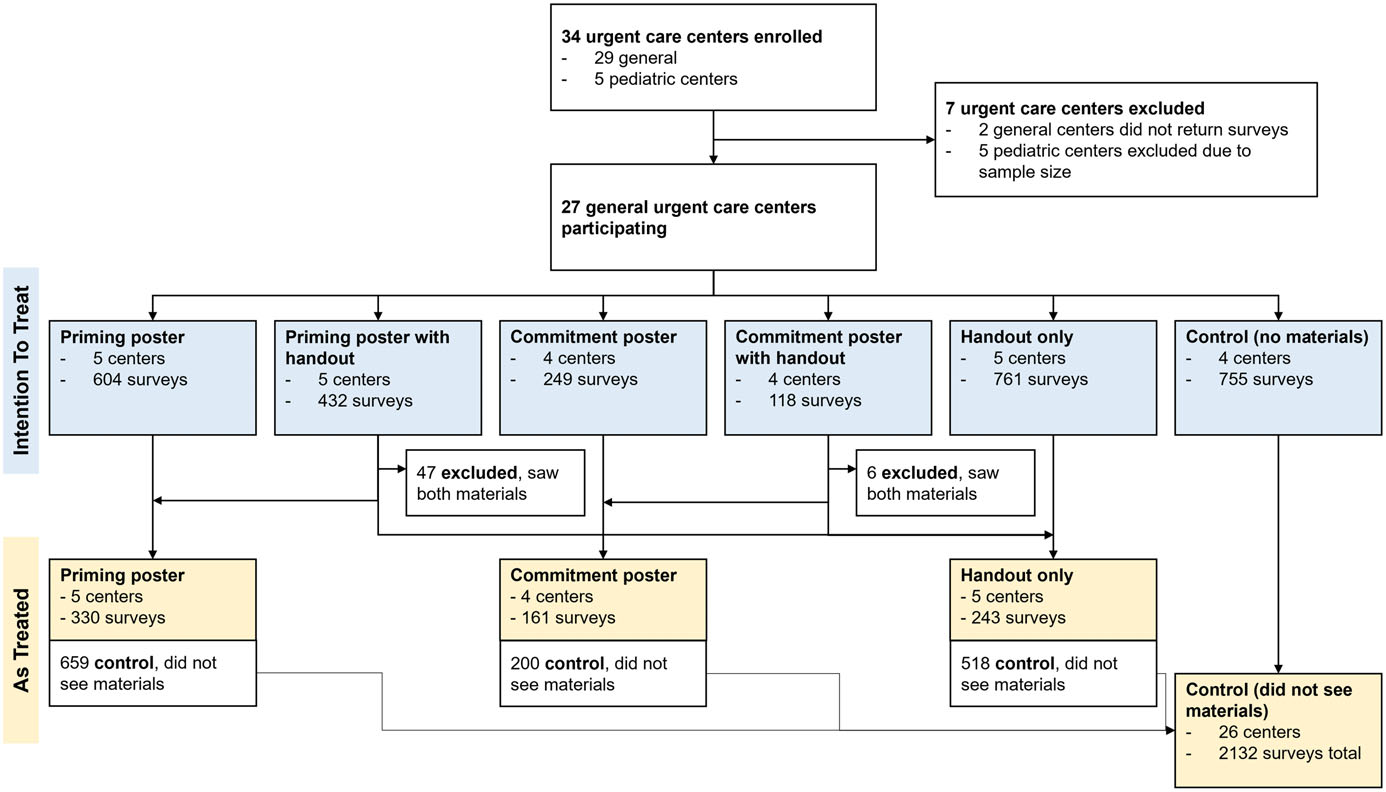
Flowchart of randomization and enrollment of urgent care centers.

Completed questionnaires were received from 27 of 29 recruited UCCs, resulting in 2919 questionnaires and four to five centers per arm (Figure 1). The number of respondents differed between study arms. There were 604 in the priming poster arm, 432 in the priming poster with handout arm, 249 in the commitment poster arm, 118 respondents in the commitment poster with handout arm, 761 in the handout arm, and 755 in the control arm (Figure 1). Study participants’ socio-demographic characteristics also varied significantly by study arm (Table 1). Across all arms, most participants were White (range: 69.5%–86.3%) and female (range: 62.4%–70.6%). Control arm participants tended to be older, with 52.6% being over 50 years of age, while 33.1% in the commitment poster with handout arm were over 50 years of age.

**Table 1.** Distribution of study arms by demographic and potential confounding variables

	Clinic Arm, n (%)						
	Priming (n = 604)	Priming + Handout (n = 432)	Commitment (n = 249)	Commitment + Handout (n = 118)	Handout (n = 761)	Control (n = 755)	*P*-value
Recalled Materials							<.001
Correct materials only	175 (29.0)	30 (6.9)	125 (50.2)	5 (4.2)	128 (16.8)	680 (90.1)	
Correct with incorrect	52 (8.6)	17 (3.9)	12 (4.8)	1 (0.9)	43 (5.7)	–	
No materials	345 (57.1)	219 (50.7)	104 (41.8)	56 (47.5)	533 (70.0)	–	
At least one correct	–	130 (30.1)	–	42 (35.6)	–	–	
Incorrect only	32 (5.3)	36 (8.3)	8 (3.2)	14 (11.9)	57 (7.5)	75 (9.9)	
Gender							.013
Male	199 (33.0)	121 (28.0)	78 (31.3)	33 (28.0)	235 (30.9)	267 (35.4)	
Female	401 (66.4)	305 (70.6)	165 (66.3)	82 (69.5)	516 (67.8)	471 (62.4)	
Other	1 (0.2)	0 (0.0)	0 (0.0)	2 (1.7)	3 (0.4)	1 (0.1)	
Did not Answer	3 (0.5)	6 (1.4)	6 (2.4)	1 (0.9)	7 (0.9)	16 (2.1)	
Age group							< .001
18–29 years	133 (22.0)	74 (17.1)	34 (13.7)	24 (20.3)	194 (25.5)	130 (17.2)	
30–49 years	237 (39.2)	135 (31.3)	100 (40.2)	54 (45.8)	235 (30.9)	213 (28.2)	
50–64 years	126 (20.9)	119 (27.6)	55 (22.1)	27 (22.9)	179 (23.5)	205 (27.2)	
≥ 65 years	104 (17.2)	100 (23.2)	53 (21.3)	12 (10.2)	146 (19.2)	192 (25.4)	
Did not answer	4 (0.7)	4 (0.9)	7 (2.8)	1 (0.9)	7 (0.9)	15 (2.0)	
Ethnicity							.008
Hispanic	65 (10.8)	34 (7.9)	23 (9.2)	21 (17.8)	70 (9.2)	56 (7.4)	
Non-Hispanic	530 (87.7)	387 (89.6)	213 (85.5)	93 (78.8)	671 (88.2)	673 (89.1)	
Did not answer	9 (1.5)	11 (2.6)	13 (5.2)	4 (3.4)	20 (2.6)	26 (3.4)	
Race							< .001
White	515 (85.3)	373 (86.3)	196 (78.7)	82 (69.5)	657 (86.3)	640 (84.8)	
Black	28 (4.6)	23 (5.3)	20 (8.0)	18 (15.3)	22 (2.9)	47 (6.2)	
Other or multiple	45 (7.5)	23 (5.3)	8 (3.2)	5 (4.2)	58 (7.6)	33 (4.4)	
Chose not to share	12 (2.0)	11 (2.6)	14 (5.6)	9 (7.6)	14 (1.8)	16 (2.1)	
Did not answer	4 (0.7)	2 (0.5)	11 (4.4)	4 (3.4)	10 (1.3)	19 (2.5)	
Education							< .001
< HS Diploma/ GED	8 (1.3)	16 (3.7)	5 (2.0)	3 (2.5)	15 (2.0)	15 (2.0)	
HS Diploma/ GED	166 (27.5)	153 (35.4)	75 (30.1)	33 (28.0)	239 (31.4)	195 (25.8)	
Trade/ associate’s degree	106 (17.6)	106 (24.5)	59 (23.7)	16 (13.6)	145 (19.1)	177 (23.4)	
Bachelor’s degree	187 (31.0)	101 (23.4)	60 (24.1)	33 (28.0)	210 (27.6)	223 (29.5)	
Master’s degree	91 (15.1)	34 (7.9)	25 (10.0)	15 (12.7)	85 (11.2)	83 (11.0)	
Doctoral/ professional degree	31 (5.1)	13 (3.0)	8 (3.2)	9 (7.6)	51 (6.7)	29 (3.8)	
Did not answer	15 (2.5)	9 (2.1)	17 (6.8)	9 (7.6)	16 (2.1)	33 (4.4)	

### Patient recall of educational materials

Of the participants assigned to an intervention arm, 1,257 of 2,164 (58.1%) participants reported not seeing any materials during their visit (Table 1). Only 588 (27.2%) participants correctly recalled seeing materials that were assigned to their clinic. Of the 588 participants who correctly recalled seeing materials assigned to their clinic, 125 (5.8%) also reported seeing materials that were not assigned to their clinic. Therefore, only 463 (21.4%) participants correctly identified the materials assigned to their clinic without also reporting incorrect materials. Some participants in the control arm (9.9%) reported seeing a material in their center despite there not being one. Poor observance of materials occurred at nearly all centers. Even among the top quartile of UCCs with the highest recall rates of the materials, less than half of participants (40.9%) were able to correctly recall seeing the educational materials. In the UCCs with the lowest recall rates (bottom quartile), only 8.5% of participants correctly recalled seeing the materials. Knowledge scores were higher among participants who correctly recalled seeing materials, compared with participants who did not see correct materials (*P* = .029); there were no differences in expectation scores based on recall of materials (*P* = .17) (Table 2).

**Table 2.** Knowledge and expectation score means^a^ by demographic and potential confounding variables

	Knowledge score mean (SD)	*P*-value	Expectation score mean (SD)	*P*-value
Recalled seeing materials		.029		.167
Correct materials only	2.41 (1.04)		4.71 (1.75)	
Correct with incorrect	2.35 (1.07)		5.07 (1.72)	
No materials	2.27 (1.06)		4.80 (1.77)	
Incorrect only	2.09 (1.13)		4.54 (2.31)	

a Fully adjusted model accounts for clinic, gender, age group, ethnicity, race, and education level.

### Impact of educational materials on antibiotic knowledge and expectations

In the ITT analysis, although there were differences between study arms in the unadjusted model, there were no significant associations between study arms when accounting for clustering by clinic. In the unadjusted model, knowledge scores were significantly different between groups (*P* = .004), with the lowest scores in the control arm (mean = 2.11, SD = 1.13), and higher knowledge scores in the priming poster (mean = 2.32, SD = 1.08, *P* < .001) and handout-only arms (mean = 2.29, SD = 1.05, *P* = .001) (Table S3). Expectation scores were not significantly different between arms (*P* = .71) (Table S3).

**Figure 2.**
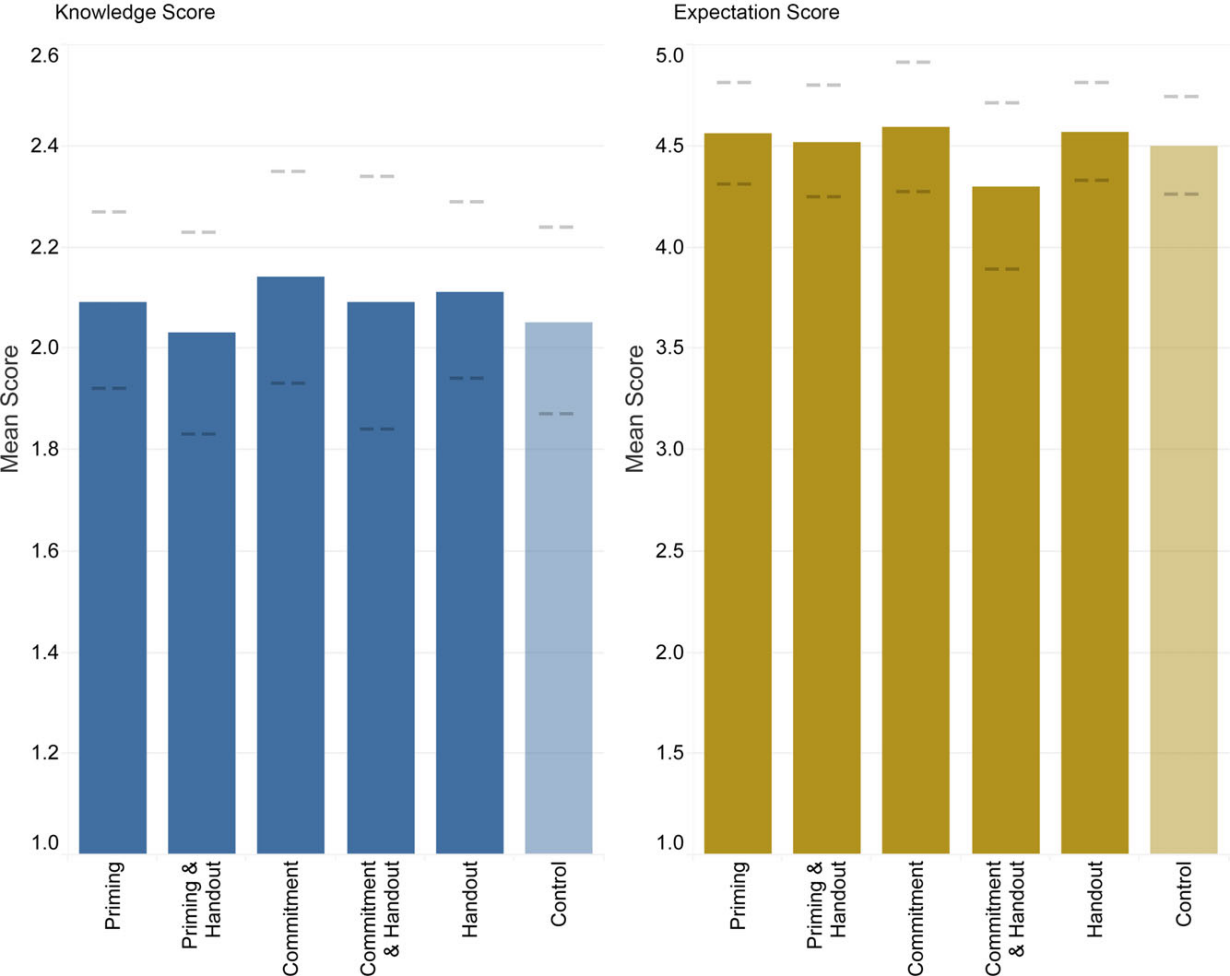
Intention-to-treat comparison of knowledge and expectation scores among each study arm (material group) vs control (assigned no materials), clustered by clinic and adjusted for gender, age group, ethnicity, race, and education level. 95% confidence intervals for each score indicated by dashed lines.

When controlling for clustering by clinic using a mixed-effects model, there were no significant associations in knowledge scores or expectation scores by group (Figure 2).

### As-treated analysis of educational materials and antibiotic knowledge and expectations

After excluding all participants who saw more than one intervention from the two interaction arms, 2,866 participants remained eligible for the as-treated analysis. In both the unadjusted and adjusted regression models, both the commitment poster and handout were associated with higher knowledge scores. In the unadjusted analysis, compared with participants who did not see the materials (mean score = 2.16), higher knowledge scores were noted for participants who saw the commitment poster (10.6% higher, mean score = 2.39, *P* = .039), handout (7.9% higher, mean score = 2.33, *P* = .033), and priming poster (7.4% higher, mean score = 2.32, *P* = .024) (Table S4).

**Figure 3.**
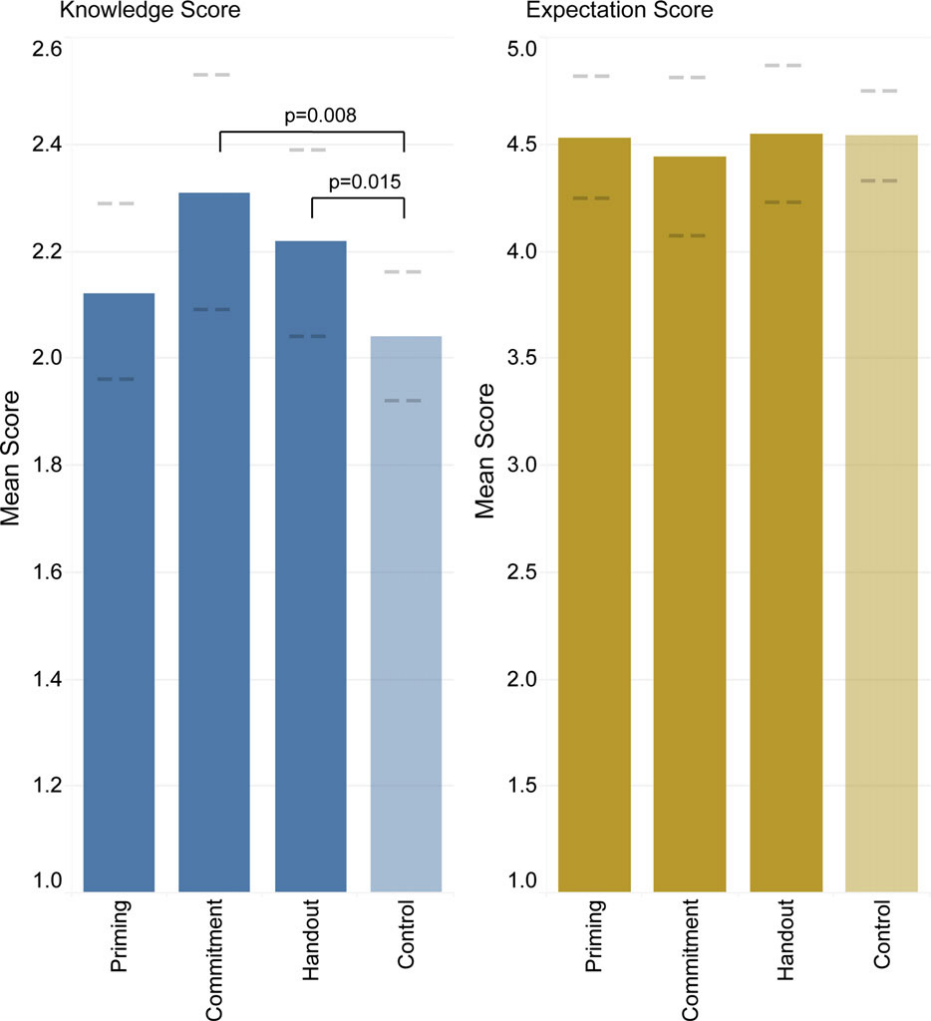
As-treated analysis of knowledge and expectation scores for participants who recalled seeing each intervention compared to control (did not recall seeing materials), clustered by clinic and adjusted for gender, age group, ethnicity, race, and education level. 95% confidence intervals for each score indicated by dashed lines.

When controlling for clustering by clinic and adjusted for covariates, both the handout and commitment poster remained associated with higher knowledge scores (8.8% higher, *P* = .02 and 13.2% higher, *P* = 0.01, respectively), but not the priming poster. The educational interventions were not associated with differences in patient expectation scores in either the unadjusted or adjusted model (Figure 3). In supplemental analyses, when limiting to diagnoses typically inappropriate for antibiotic treatment, inappropriate expectation scores tended to be lower among the handout and commitment poster arms, but differences were not statistically significant (7% lower, *P* = .16 and 11% lower, *P* = .13, respectively) (Table S4). Knowledge scores were significantly inversely associated with expectation scores (*ρ* = –.13, *P* < .001) and inappropriate expectation scores (*ρ* = –.42, *P* < 0.001).

## Discussion

This multi-state, multi-site block randomized study of over 2,000 patients at U.S. UCCs evaluated the effectiveness of multiple educational materials on improving patients’ antibiotic knowledge and expectations. While educational materials can improve patient knowledge regarding antibiotic prescribing, most urgent care patients do not notice passively displayed educational materials, highlighting the challenge of implementing and studying health promotional interventions in the urgent care setting.

Participants at nearly all UCCs showed poor recall of the material(s) that was/were displayed in their care setting, suggesting that posters and handouts may be ineffective interventions across a variety of urgent care settings. Poor recall may explain the limited impact of education material(s) on participant antibiotic knowledge or antibiotic expectation scores in the ITT analysis. As a result, we also could not evaluate additive or multiplicative interaction of the materials in the two-material arms. After controlling for covariates, the as-treated analysis showed that participants who correctly recalled seeing the material had higher antibiotic knowledge scores compared to those who did not recall seeing the material, while there were no differences in patient antibiotic expectation scores.

Prior studies on patient education for antimicrobial stewardship have had mixed results, potentially also due to patients not noticing the materials.^13–17^ A single center study in a family practice in the northeastern United States evaluating the effect of posters, GIFs, and memes displayed in waiting rooms reported a 12.6% decrease in antibiotic prescribing, with anecdotal notes that GIFs and memes had drawn patient attention and may have bolstered effectiveness of patient education interventions.^14^ Factors such as competing health messaging materials in the waiting room, participants who are coping with acute illness, and commonness of phone usage in the waiting room may help to explain why participants were significantly more likely to notice educational materials in the exam room than in the waiting room. While stewardship interventions do not typically assess recall of patient-directed materials, our study quantifies how the effectiveness of patient educational materials may largely be determined by whether or not patients notice the materials. Our findings highlight the low rates of recall across a large, diverse set of urgent care centers, suggesting that simple efforts to increase awareness of materials could significantly improve effectiveness of patient-directed stewardship interventions.

In contrast to improved antibiotic knowledge, there was no effect on the patient antibiotic expectation scores, which may be a result of residual confounding. Control arm participants reported higher educational levels, which is generally associated with lower antibiotic expectations.^17^ The antibiotic expectation questions included both antibiotic-appropriate and inappropriate conditions and better knowledge of antibiotic appropriateness may have resulted in both lower responses for inappropriate conditions and higher responses for appropriate conditions, leading to a similar total score. For example, the handout arm had lower antibiotic expectations for colds (*P* = .08) but higher expectations for strep throat (*P* < .001), as compared to the control arm. Supplemental analyses restricting to antibiotic-inappropriate diagnoses showed decreased expectations in the intervention arms, but the differences were not statistically significant. Consistent with prior studies, antibiotic knowledge and expectation were inversely correlated.^24^

Our study had limitations. Participants with better recall of information may be inherently different from participants who see but are unable to recall interventions. Similarly, the study may have response bias where participants who responded may be more likely to participate depending on their antibiotic related knowledge or expectations. There may be misclassification in the as-treated analysis including participants who did not see the materials but reported having seen them, and vice versa. Additional misclassification or data validity issues may result from data entry errors or through the anonymous self-reporting approach; such non-differential misclassification is likely to bias findings towards the null. Sensitivity analyses that only included participants who correctly recalled seeing the materials without reporting seeing any other materials, compared with participants who did not see any materials, were consistent with the main as-treated analysis. Based on simulation studies with cluster-randomized trials,^25^ our ITT analysis may be underpowered to detect statistically significant differences for small effect sizes. Enrolling UCC sites from multiple states provided many benefits, including increased sample size and improved generalizability across US regions; however, it limited our ability to assess compliance with display of materials at each site, and we were not able to collect the number of patients seen at each clinic to evaluate potential differential participation. The UCCs, including those in the control arm, may have had existing antibiotic stewardship interventions in place. Although we did not capture this information directly, no other materials were noted in setup pictures provided by UCCs or noted by UCCs directly. While the results of the as-treated analysis indicate statistically significant differences in scores for some of the interventions, this does not necessarily imply that there is clinical significance. Lastly, while commitment posters as reported by Meeker et al. are shown to improve antibiotic prescribing,^22^ our study focused on patient-level knowledge and expectations and did not assess impact on providers or rates of inappropriate prescribing.

Overall, the current study assessed how educational materials displayed in UCCs impacted patient antibiotic-related knowledge and expectations, which showed that few participants could recall the displayed materials and that there was no significant passive benefit. Patient educational interventions that require passive observation in UCCs show limited utility if they are not seen. However, participants who recalled the material displayed in their clinic had improved antibiotic-related knowledge compared to participants who did not see the materials, indicating the importance of materials that can capture patients’ attention. Future research should assess factors that promote uptake of educational materials in outpatient settings, in addition to evaluating overall effectiveness of the interventions.

## Supporting information

Cziner et al. supplementary material Cziner et al. supplementary material

## Data Availability

The data are available from the corresponding author on reasonable request.

## Supplementary material

For supplementary material accompanying this paper visit http://doi.org/10.1017/ash.2024.475. click here to view supplementary material
