# Supplementary material for: Efficacy of priming and commitment posters on urgent care patients’ antibiotic expectations and knowledge: a cluster randomized trial
Source: Antimicrob Steward Healthc Epidemiol. 2025 Jan 6;5(1):e1. doi: 10.1017/ash.2024.475 (PMC11704940; doi:10.1017/ash.2024.475)
Supplement: Cziner et al. supplementary material [file S2732494X24004753sup001.docx]

**Supplementary Materials**

**Efficacy of Priming and Commitment Posters on Urgent Care Patients’ Antibiotic Expectations and Knowledge: A Cluster Randomized Trial**

**Table S1. Knowledge and expectation score means by demographic and potential confounding variables**

|  | **Knowledge Score Mean (SD)** | **p-value** | **Expectation Score Mean (SD)** | **p-value** |
| --- | --- | --- | --- | --- |
| **Gender** |  | < 0.001 |  | 0.958 |
| Male | 2.13 (1.13) |  | 4.75 (2.04) |  |
| Female | 2.31 (1.05) |  | 4.75 (1.76) |  |
| Other | 2.20 (2.05) |  | 6.40 (2.51) |  |
| Did not Answer | 1.72 (1.14) |  | 5.00 (1.80) |  |
| **Age Group** |  | < 0.001 |  | < 0.001 |
| 18-29 years | 2.15 (1.13) |  | 5.19 (2.02) |  |
| 30-49 years | 2.35 (1.08) |  | 4.80 (1.71) |  |
| 50-64 years | 2.35 (1.01) |  | 4.63 (1.76) |  |
| ≥ 65 years | 2.09 (1.08) |  | 4.39 (1.93) |  |
| Did not Answer | 1.85 (1.08) |  | 4.82 (1.54) |  |
| **Ethnicity** |  | 0.053 |  | 0.595 |
| Hispanic | 2.13 (1.11) |  | 4.83 (2.05) |  |
| Non-Hispanic | 2.28 (1.07) |  | 4.76 (1.83) |  |
| Did not Answer | 1.65 (1.13) |  | 4.38 (2.11) |  |
| **Race** |  | < 0.001 |  | 0.160 |
| White | 2.29 (1.08) |  | 4.74 (1.82) |  |
| Black | 1.93 (1.07) |  | 5.11 (2.13) |  |
| Other or Multiple | 2.20 (1.05) |  | 4.62 (1.91) |  |
| Chose not to Share | 1.98 (1.05) |  | 4.88 (2.19) |  |
| Did not Answer | 1.56 (1.11) |  | 4.80 (2.21) |  |
| **Education** |  | < 0.001 |  | < 0.001 |
| < HS Diploma/ GED | 1.35 (1.19) |  | 6.00 (2.12) |  |
| HS Diploma/ GED | 2.00 (1.05) |  | 4.93 (2.01) |  |
| Trade/ Associate's Degree | 2.18 (1.05) |  | 4.78 (1.93) |  |
| Bachelor's Degree | 2.44 (1.06) |  | 4.70 (1.70) |  |
| Master's Degree | 2.60 (0.93) |  | 4.38 (1.60) |  |
| Doctoral/ Professional Degree | 2.71 (1.07) |  | 4.42 (1.52) |  |
| Did not Answer | 1.59 (1.10) |  | 4.79 (2.18) |  |

**Table S2. Knowledge and expectation scores and distribution of score components by arm**

|  | Clinic Arm, n (%) | | | | | |  |
| --- | --- | --- | --- | --- | --- | --- | --- |
|  | Priming + Handout (n = 432) | Commitment + Handout (n = 118) | Handout (n = 761) | Priming (n = 604) | Commitment (n = 249) | Control (n = 755) | p-value^a^ |
| **Knowledge Score, Mean (SD)** | 2.13 (1.09) | 2.28 (1.08) | 2.29 (1.05) | 2.32 (1.10) | 2.24 (1.07) | 2.11 (1.13) | 0.004 |
| **Q6) What do antibiotics help treat?** | | | | | | | 0.527 |
| Cold/Flu | 20 (4.6) | 12 (10.2) | 22 (2.9) | 20 (3.3) | 13 (5.2) | 35 (4.6) |  |
| Virus | 22 (5.1) | 1 (0.9) | 28 (3.7) | 23 (3.8) | 14 (5.6) | 44 (5.8) |  |
| Bacteria | 290 (67.1) | 83 (70.3) | 539 (70.8) | 436 (72.2) | 165 (66.3) | 505 (66.9) |  |
| Cold/Flu & Virus | 0 (0.0) | 0 (0.0) | 3 (0.4) | 3 (0.5) | 1 (0.4) | 3 (0.4) |  |
| Cold/Flu & Bacteria | 19 (4.4) | 0 (0.0) | 24 (3.2) | 18 (3.0) | 8 (3.2) | 33 (4.4) |  |
| Virus & Bacteria | 23 (5.3) | 7 (5.9) | 41 (5.4) | 37 (6.1) | 14 (5.6) | 33 (4.4) |  |
| All of the Above | 29 (6.7) | 5 (4.2) | 53 (7.0) | 30 (5.0) | 17 (6.8) | 43 (5.7) |  |
| None of the Above | 13 (3.0) | 9 (7.6) | 34 (4.5) | 23 (3.8) | 9 (3.6) | 32 (4.2) |  |
| Did Not Answer | 16 (3.7) | 1 (0.9) | 17 (2.2) | 14 (2.3) | 8 (3.2) | 27 (3.6) |  |
| **Q7) If a doctor is unsure if an antibiotic is needed, should one be prescribed just in case?** | | | | | | | 0.101 |
| True | 43 (10.0) | 17 (14.4) | 85 (11.2) | 58 (9.6) | 24 (9.6) | 94 (12.5) |  |
| False | 277 (64.1) | 79 (67.0) | 511 (67.2) | 420 (69.5) | 180 (72.3) | 467 (61.9) |  |
| I don't know | 105 (24.3) | 21 (17.8) | 148 (19.5) | 115 (19.0) | 42 (17.0) | 175 (23.2) |  |
| Did Not Answer | 7 (1.6) | 1 (0.9) | 17 (2.2) | 11 (1.8) | 3 (1.2) | 19 (2.5) |  |
| **Q8) Are antibiotic resistant infections due to bacteria resistant to antibiotics** | | | | | | | 0.147 |
| True | 296 (68.5) | 83 (70.3) | 564 (74.1) | 435 (72.0) | 174 (69.9) | 510 (67.6) |  |
| False | 26 (6.0) | 8 (6.8) | 49 (6.4) | 43 (7.1) | 15 (6.0) | 45 (6.0) |  |
| I don't know | 105 (24.3) | 25 (21.2) | 133 (17.5) | 115 (19.0) | 56 (22.5) | 183 (24.2) |  |
| Did Not Answer | 5 (1.2) | 2 (1.7) | 15 (2.0) | 11 (1.8) | 4 (1.6) | 17 (2.3) |  |
| **Q10) What increases risk of becoming infected by bacteria that are resistance to antibiotics?** | | | | | | | 0.087 |
| Taking an antibiotic can make a person resistant to antibiotics | 33 (7.6) | 8 (6.8) | 50 (6.6) | 49 (8.1) | 19 (7.6) | 43 (5.7) |  |
| Taking an antibiotic can make bacteria resistant to antibiotics | 57 (13.2) | 24 (20.3) | 129 (17.0) | 108 (17.9) | 40 (16.1) | 111 (14.7) |  |
| Both | 196 (45.4) | 45 (38.1) | 351 (46.1) | 275 (45.5) | 98 (39.4) | 328 (43.4) |  |
| I don't know | 130 (30.1) | 37 (31.4) | 200 (26.3) | 150 (24.8) | 75 (30.1) | 239 (31.7) |  |
| Did Not Answer | 16 (3.7) | 4 (3.4) | 31 (4.1) | 22 (3.6) | 17 (6.8) | 34 (4.5) |  |
| **Expectation Score, Mean (SD)** | 4.63 (1.91) | 4.46 (2.20) | 4.63 (1.96) | 4.60 (1.92) | 4.62 (2.10) | 4.40 (2.23) | 0.712 |
| **Q11) What do you expect an antibiotic for?** | | | | | | |  |
| **Cold** | 64 (14.8) | 22 (18.6) | 107 (14.1) | 74 (12.3) | 38 (15.3) | 131 (17.4) | 0.009 |
| **Cough** | 88 (20.4) | 23 (19.5) | 135 (17.7) | 108 (17.9) | 43(17.3) | 173 (22.9) | < 0.001 |
| **Virus** | 116 (26.9) | 25 (21.2) | 200 (26.3) | 130 (21.5) | 62 (24.9) | 174 (23.1) | < 0.001 |
| **Strep Throat** | 357 (82.6) | 89 (75.4) | 649 (85.3) | 508 (84.1) | 206 (82.7) | 585 (77.5) | < 0.001 |
| **Flu** | 125 (28.9) | 27 (22.9) | 199 (26.2) | 166 (27.5) | 73 (29.3) | 217 (28.7) | 0.007 |
| **UTI** | 361 (83.6) | 96 (81.4) | 656 (86.2) | 514 (85.1) | 208 (83.5) | 600 (79.5) | 0.003 |
| **Bronchitis** | 308 (71.3) | 73 (61.9) | 518 (68.1) | 413 (68.4) | 175 (70.3) | 498 (66.0) | 0.042 |
| **Skin Infection** | 255 (59.0) | 84 (71.2) | 496 (65.2) | 388 (64.2) | 153 (61.5) | 423 (56.0) | 0.001 |
| **Sinus Infection** | 326 (75.5) | 87 (73.7) | 565 (74.2) | 478 (79.1) | 193 (77.5) | 519 (68.7) | < 0.001 |

a. comparing single intervention arms

**Table S3. Intention-to-Treat comparison of a) knowledge scores, b) total expectation scores, and c) inappropriate expectation scores among each study arm (material group) to control (no materials), using a generalized linear mixed model clustered by clinic.**

| **a. Knowledge Score** | **Unadjusted** | | | | | **Adjusted*** | | | | |
| --- | --- | --- | --- | --- | --- | --- | --- | --- | --- | --- |
| **Material** | **N** | **Mean (95% CI)** | **SE** | **Estimate** | **p-value** | **N** | **Mean (95% CI)** | **SE** | **Estimate** | **p-value** |
| Priming | 604 | 2.26 (2.08-2.45) | 0.09 | 0.06 | 0.67 | 579 | 2.09 (1.92-2.27) | 0.09 | 0.04 | 0.71 |
| Handout + Priming | 432 | 2.14 (1.91-2.36) | 0.11 | -0.07 | 0.66 | 413 | 2.03 (1.83-2.23) | 0.10 | -0.02 | 0.84 |
| Commitment | 249 | 2.18 (1.96-2.40) | 0.11 | -0.02 | 0.88 | 226 | 2.14 (1.93-2.35) | 0.11 | 0.09 | 0.48 |
| Handout + Commitment | 118 | 2.27 (2.01-2.53) | 0.13 | 0.06 | 0.70 | 102 | 2.09 (1.84-2.34) | 0.13 | 0.03 | 0.81 |
| Handout | 761 | 2.27 (2.09-2.45) | 0.09 | 0.07 | 0.62 | 727 | 2.11 (1.94-2.29) | 0.09 | 0.06 | 0.58 |
| Control | 755 | 2.20 (2.00-2.40) | 0.10 | Ref | –– | 703 | 2.05 (1.87-2.24) | 0.09 | Ref | –– |

| **b. Expectation Score** | **Unadjusted** | | | | | **Adjusted*** | | | | |
| --- | --- | --- | --- | --- | --- | --- | --- | --- | --- | --- |
| **Material** | **N** | **Mean (95% CI)** | **SE** | **Estimate** | **p-value** | **N** | **Mean (95% CI)** | **SE** | **Estimate** | **p-value** |
| Priming | 604 | 4.60 (4.43-4.78) | 0.09 | 0.20 | 0.11 | 579 | 4.56 (4.31-4.81) | 0.13 | 0.06 | 0.58 |
| Handout + Priming | 432 | 4.64 (4.43-4.85) | 0.11 | 0.23 | 0.09 | 413 | 4.52 (4.25-4.80) | 0.14 | 0.03 | 0.84 |
| Commitment | 249 | 4.63 (4.37-4.90) | 0.13 | 0.22 | 0.15 | 226 | 4.59 (4.27-4.91) | 0.16 | 0.09 | 0.54 |
| Handout + Commitment | 118 | 4.46 (4.09-4.84) | 0.19 | 0.06 | 0.79 | 102 | 4.30 (3.89-4.71) | 0.21 | -0.20 | 0.34 |
| Handout | 761 | 4.63 (4.47-4.79) | 0.08 | 0.23 | 0.05 | 727 | 4.57 (4.33-4.81) | 0.12 | 0.07 | 0.49 |
| Control | 755 | 4.41 (4.24-4.57) | 0.08 | Ref | - | 703 | 4.50 (4.26-4.74) | 0.12 | Ref | - |

| **c. Inappropriate Expectation Score** | **Unadjusted** | | | | | **Adjusted*** | | | | |
| --- | --- | --- | --- | --- | --- | --- | --- | --- | --- | --- |
| **Material** | **N** | **Mean (95% CI)** | **SE** | **Estimate** | **p-value** | **N** | **Mean (95% CI)** | **SE** | **Estimate** | **p-value** |
| Priming | 604 | 1.59 (1.34-1.84) | 0.13 | -0.10 | 0.57 | 579 | 1.78 (1.52-2.04) | 0.13 | -0.08 | 0.63 |
| Handout + Priming | 432 | 1.69 (1.39-1.98) | 0.15 | -0.01 | 0.96 | 413 | 1.80 (1.50-2.10) | 0.15 | -0.06 | 0.73 |
| Commitment | 249 | 1.71 (1.41-2.00) | 0.15 | 0.01 | 0.96 | 226 | 1.80 (1.50-2.11) | 0.15 | -0.06 | 0.76 |
| Handout + Commitment | 118 | 1.59 (1.25-1.93) | 0.17 | -0.11 | 0.62 | 102 | 1.70 (1.35-2.05) | 0.18 | -0.16 | 0.44 |
| Handout | 761 | 1.59 (1.35-1.84) | 0.12 | -0.10 | 0.58 | 727 | 1.76 (1.50-2.01) | 0.13 | -0.11 | 0.52 |
| Control | 755 | 1.70 (1.43-1.96) | 0.14 | Ref | - | 703 | 1.86 (1.59-2.13) | 0.14 | Ref | - |

* Fully adjusted model accounts for clinic arm, gender, age group, ethnicity, race, and education level

**Table S4. As-treated analysis of a) knowledge scores, b) total expectation scores, and c) inappropriate expectation scores for participants who recalled seeing each intervention compared to control (did not recall seeing materials), using a generalized linear mixed model clustered by clinic.**

| **a. Knowledge Score** | **Unadjusted** | | | | | **Adjusted*** | | | | |
| --- | --- | --- | --- | --- | --- | --- | --- | --- | --- | --- |
| **Material** | **N** | **Mean (95% CI)** | **SE** | **Estimate** | **p-value** | **N** | **Mean (95% CI)** | **SE** | **Estimate** | **p-value** |
| Priming | 330 | 2.32 (2.18-2.47) | 0.07 | 0.16 | **0.024** | 320 | 2.12 (1.96-2.29) | 0.08 | 0.09 | 0.182 |
| Commitment | 161 | 2.39 (2.18-2.60) | 0.11 | 0.23 | **0.039** | 146 | 2.31 (2.09-2.53) | 0.11 | 0.27 | **0.008** |
| Handout | 243 | 2.33 (2.17-2.49) | 0.08 | 0.16 | **0.033** | 230 | 2.22 (2.04-2.39) | 0.09 | 0.18 | **0.015** |
| Control | 2132 | 2.16 (2.08-2.25) | 0.04 | Ref | - | 2001 | 2.04 (1.92-2.16) | 0.06 | Ref | - |

| **b. Expectation Score** | **Unadjusted** | | | | | **Adjusted*** | | | | |
| --- | --- | --- | --- | --- | --- | --- | --- | --- | --- | --- |
| **Material** | **N** | **Mean (95% CI)** | **SE** | **Estimate** | **p-value** | **N** | **Mean (95% CI)** | **SE** | **Estimate** | **p-value** |
| Priming | 330 | 4.62 (4.39-4.85) | 0.12 | 0.07 | 0.55 | 320 | 4.53 (4.25-4.82) | 0.15 | -0.004 | 0.98 |
| Commitment | 161 | 4.59 (4.26-4.92) | 0.17 | 0.04 | 0.82 | 146 | 4.44 (4.07-4.81) | 0.19 | -0.10 | 0.55 |
| Handout | 243 | 4.65 (4.38-4.91) | 0.14 | 0.10 | 0.46 | 230 | 4.55 (4.23-4.87) | 0.16 | 0.01 | 0.92 |
| Control | 2132 | 4.55 (4.45-4.65) | 0.05 | Ref | - | 2001 | 4.54 (4.33-4.75) | 0.11 | Ref | - |

| **c. Inappropriate Expectation Score** | **Unadjusted** | | | | | **Adjusted*** | | | | |
| --- | --- | --- | --- | --- | --- | --- | --- | --- | --- | --- |
| **Material** | **N** | **Mean (95% CI)** | **SE** | **Estimate** | **p-value** | **N** | **Mean (95% CI)** | **SE** | **Estimate** | **p-value** |
| 0.01Priming | 330 | 1.56 (1.37-1.74) | 0.09 | -0.12 | 0.17 | 320 | 1.75 (1.53-1.97) | 0.11 | -0.08 | 0.34 |
| Commitment | 161 | 1.60 (1.33-1.87) | 0.14 | -0.08 | 0.56 | 146 | 1.63 (1.33-1.92) | 0.15 | -0.21 | 0.13 |
| Handout | 243 | 1.59 (1.39-1.79) | 0.10 | -0.09 | 0.35 | 230 | 1.70 (1.47-1.93) | 0.12 | -0.13 | 0.16 |
| Control | 2132 | 1.68 (1.57-1.79) | 0.06 | Ref | - | 2001 | 1.83 (1.67-2.00) | 0.09 | Ref | - |

* Fully adjusted model accounts for clinic arm, gender, age group, ethnicity, race, and education level

**Figure S1. Posters Displayed in Participating Urgent Care Centers**

| 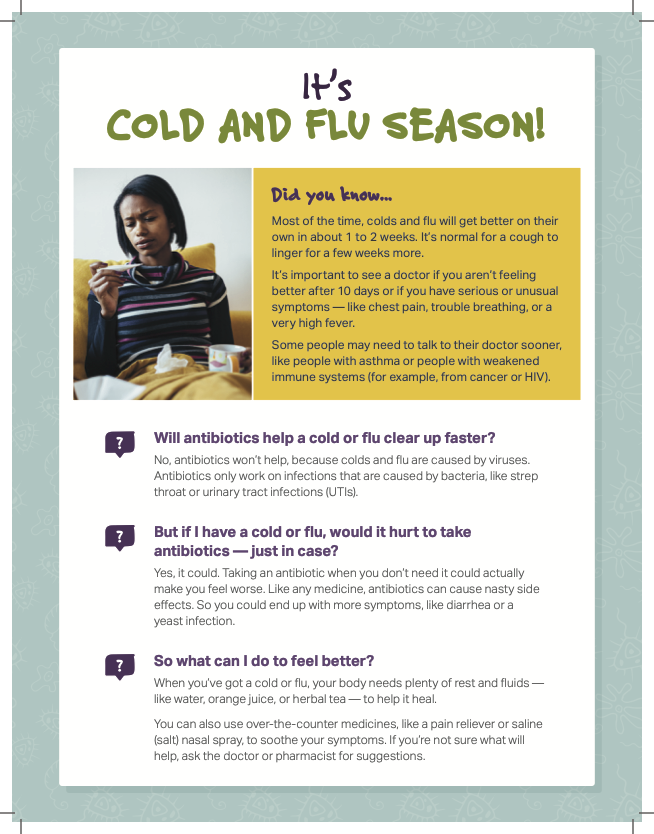**Handout (Front)** | 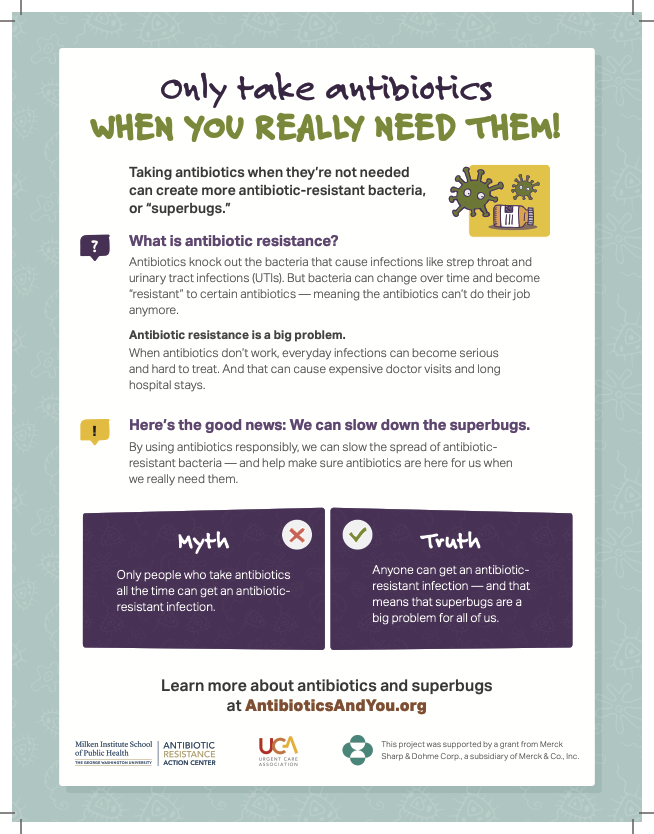**Handout (Back)** |
| --- | --- |
| 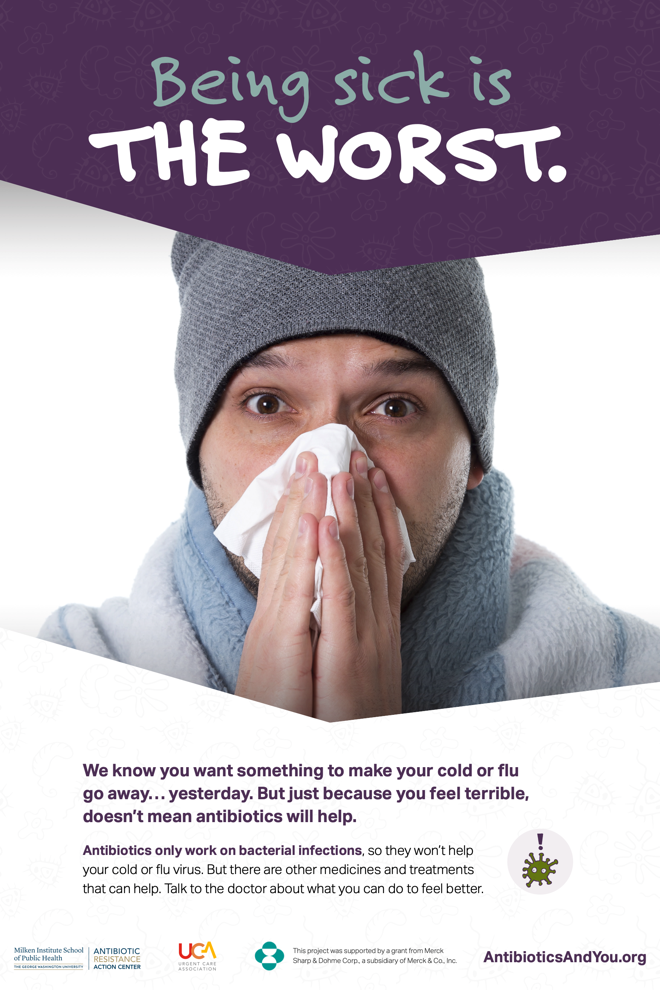**Priming Poster** | 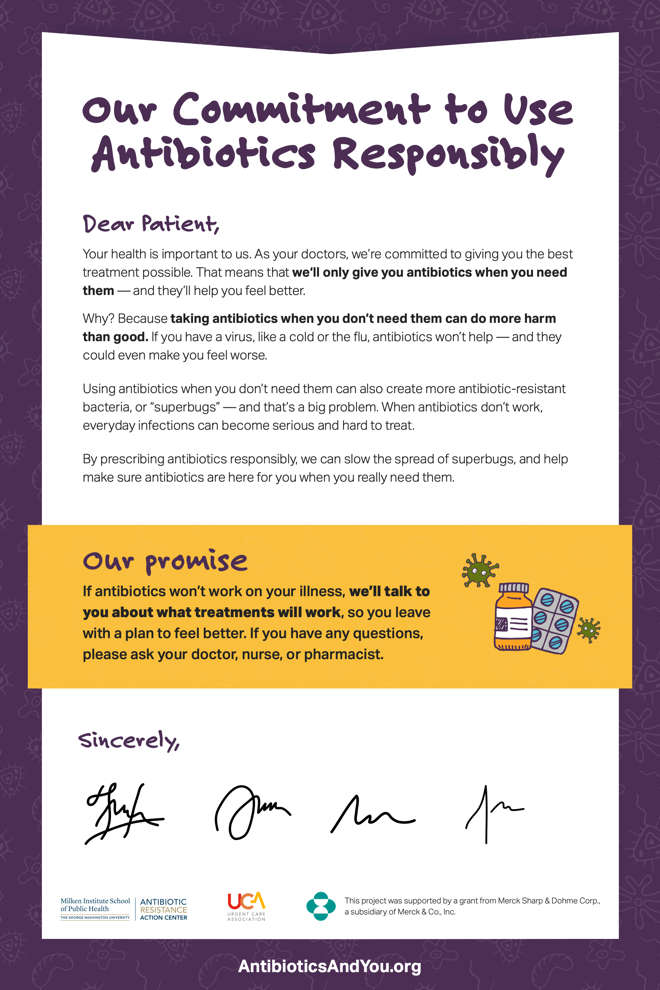**Commitment Poster** |
